# Wastewater network infrastructure in public health: Applications and learnings from the COVID-19 pandemic

**DOI:** 10.1371/journal.pgph.0000061

**Published:** 2021-12-02

**Authors:** Nour Sharara, Noriko Endo, Claire Duvallet, Newsha Ghaeli, Mariana Matus, Jennings Heussner, Scott W. Olesen, Eric J. Alm, Peter R. Chai, Timothy B. Erickson

**Affiliations:** 1 Biobot Analytics, Inc., Cambridge, Massachusetts, United States of America,; 2 Department of Biological Engineering, Massachusetts Institute of Technology, Cambridge, Massachusetts, United States of America,; 3 Center for Microbiome Informatics and Therapeutics, Massachusetts Institute of Technology, Cambridge, Massachusetts, United States of America,; 4 Singapore-MIT Alliance for Research and Technology, Antimicrobial Resistance Interdisciplinary Research Group, Singapore, Singapore,; 5 Campus for Research Excellence and Technological Enterprise (CREATE), Singapore, Singapore,; 6 Broad Institute of MIT and Harvard, Cambridge, Massachusetts, United States of America,; 7 Division of Medical Toxicology, Department of Emergency Medicine, Mass General Brigham, Harvard Medical School, Boston, Massachusetts, United States of America,; 8 The Fenway Institute, Boston, Massachusetts, United States of America,; 9 The Koch Institute for Integrated Cancer Research, Massachusetts Institute of Technology, Cambridge, Massachusetts, United States of America,; 10 Division of Psychosocial Oncology and Palliative Care, Dana Farber Cancer Institute, Boston, Massachusetts, United States of America,; 11 Harvard Humanitarian Initiative, Cambridge, Massachusetts, United States of America

## Abstract

Accurate estimates of COVID-19 burden of infections in communities can inform public health strategy for the current pandemic. Wastewater based epidemiology (WBE) leverages sewer infrastructure to provide insights on rates of infection by measuring viral concentrations in wastewater. By accessing the sewer network at various junctures, important insights regarding COVID-19 disease activity can be gained. The analysis of sewage at the wastewater treatment plant level enables population-level surveillance of disease trends and virus mutations. At the neighborhood level, WBE can be used to describe trends in infection rates in the community thereby facilitating local efforts at targeted disease mitigation. Finally, at the building level, WBE can suggest the presence of infections and prompt individual testing. In this critical review, we describe the types of data that can be obtained through varying levels of WBE analysis, concrete plans for implementation, and public health actions that can be taken based on WBE surveillance data of infectious diseases, using recent and successful applications of WBE during the COVID-19 pandemic for illustration.

## Introduction

Epidemiological surveillance is centered on monitoring the incidence of infection or disease. As a first layer, this system includes syndromic surveillance which is based on standardized symptom templates to look for signals of emerging or re-emerging diseases of public health concern [1]. It relies mostly on subsets of clinical data that can be gathered from sources like emergency department visits, hospital admissions and ambulatory care centers. As a second layer, it relies on clinical and laboratory diagnostic data.

The information generated from compiling clinical data from individual cases allows for contact tracing, trend analysis and disease modeling. It can also forecast impending increases in cases, healthcare system utilization, and hospital capacity planning [2–4]. Entry into public health data repositories relies on individuals encountering the healthcare system, often well into their disease process. Despite their expansiveness, these epidemiological surveillance systems therefore have inherent biases. The COVID-19 pandemic has highlighted the importance of disease surveillance to prevent and mitigate outbreaks of infectious diseases, but it has also exposed several system-wide limitations [5].

For biological threats like the ongoing COVID-19 pandemic, public health surveillance systems have relied primarily on clinical testing of symptomatic individuals [6, 7]. Yet because of the high proportion of asymptomatic infections, fluctuating availability and access to testing, as well as stigma associated with the disease, clusters of COVID-19 disease may become widespread before they are identified by traditional public health reporting mechanisms. Relying exclusively on clinical data and population surveys therefore runs the risk of underestimating the true scale of the pandemic [8]. Additional limitations of existing surveillance systems, which presented bottlenecks in the earlier phases of the pandemic, include high cost of testing and reporting delays.

Wastewater surveillance has emerged as an important tool, complementary to existing public health surveillance systems, to monitor community-level spread of infection during the COVID-19 pandemic [9–11]. Because SARS-CoV-2, the virus responsible for COVID-19, is shed in the stool of infected individuals, monitoring wastewater networks permits visualization of disease clusters independent of symptoms and access to testing [12]. In practice, wastewater surveillance has been implemented in many communities across the United States, Europe and globally, as a community risk assessment tool to monitor population-level spread of COVID-19 and detect clusters of infections [13, 14]. This strategy has also been employed in hotspots of infections, including schools, universities, correctional settings, and nursing homes as an early warning system and supplement to in-person testing and clinical case reporting [15]. In this manuscript, we review key considerations surrounding the decision to implement public health wastewater surveillance and operational factors associated with the use of wastewater-based epidemiology for the current pandemic and future infectious diseases.

## Wastewater-based epidemiology

Wastewater-based epidemiology (WBE) leverages existing sewer infrastructure to access wastewater from which aggregated human waste data can be extracted to inform public health officials and decision makers about population health. Any biological pathogens and biomarkers of infection which are excreted in human feces or urine can therefore in theory be monitored via wastewater-based epidemiology.

Wastewater surveillance of infectious diseases has been previously deployed to detect polio-viruses as part of the global effort to eradicate polio, to avert hepatitis A virus and norovirus outbreaks, and detect the resurgence of a salmonella outbreak undetected by traditional clinical surveillance methods [16–19]. In 2013, Israeli researchers detected an outbreak of poliovirus through WBE in advance of clinical symptoms. Leveraging this early warning, the government responded swiftly with targeted vaccination efforts that effectively contained the outbreak. Beyond infectious diseases, WBE has also been deployed to measure exposure and consumption of pharmaceutical agents and illicit drugs in cities by measuring metabolites found in wastewater, and to measure antimicrobial resistance at the community level by analyzing bacterial DNA in sewage [20–25].

While SARS-CoV-2 is primarily shed in the respiratory tract, infected individuals can also shed the virus in stool making measurement of SARS-CoV-2 in wastewater feasible [26]. By measuring viral concentrations in sewage, wastewater analysis can detect one infected person in a population of thousands of individuals [27–31]. Testing wastewater at a regular frequency allows for tracking a pandemic’s geographical and temporal trends. This can be accomplished in a cost-effective, equitable manner, providing snapshots of disease activity that are independent of individuals’ socioeconomic status or access to healthcare. In recognition of the key role wastewater surveillance data could play during the pandemic to help public health officials better understand the extent of COVID-19 infections in communities, the US Centers for Disease Control and Prevention announced in August 2020 their plan to create a National Wastewater Surveillance System (NWSS) working with state, local, territorial and tribal health departments to collect data from wastewater samples [32, 33].

In the Netherlands, a COVID-19 national wastewater monitoring program was launched at the start of the pandemic with 17 wastewater treatment plants, expanded to 80 by July 2020 and currently includes 315 municipal sampling locations covering the country’s 17 million inhabitants [34]. The program is being led by the Dutch National Institute for Public Health and the Environment and the wastewater results are published on a national dashboard updated daily [35]. There are also ongoing global sewage surveillance efforts led by Denmark’s National Food Institute. In Spain, the Catalan Surveillance Network of SARS-CoV-2 in Sewage was launched in July 2020 to monitor 56 wastewater treatment plants and capture 80% of the population living in Catalonia [36]. In Australia, the state of New South Wales (NSW), which includes Sydney, launched a COVID-19 Sewage Surveillance Program in July 2020 as part of an integrated COVID-19 surveillance and control program across NSW and sampling locations were selected based on areas of concern and direction from the NSW Chief Health Officer [37].

### Applications of wastewater-based epidemiology across phases of a pandemic

Applications of wastewater-based epidemiology vary across phases of a pandemic and in response to fluctuating health system resources. Depending on the stage of a pandemic and its specific application, wastewater epidemiology can serve primarily as an early warning system, an independent data stream to monitor disease spread and evaluate the effectiveness of public health interventions, and as a complement to genomic epidemiology.

### Early warning system

Because wastewater monitoring can detect pathogens if infected individuals shed a biomarker of the pathogen in stool, regardless of their symptomatic status, it can be leveraged as an early warning system to detect the emergence of infectious diseases within a community [38–40]. This capability has been exploited during the current COVID-19 pandemic to conduct low-intensity surveillance in buildings, at-risk congregate living settings and communities. Since clinical reporting of COVID-19 disease is dependent on in-person individual testing capacity and access, wastewater analysis has foreshadowed the onset of clinical cases thereby allowing cities and governments to allocate important resources in anticipation of new surges in infection and to prevent health care systems from being overwhelmed [41]. In the Spring of 2020, as cities were still dealing with the first surge of cases in the first phase of the pandemic, some city managers set up collaborations with universities conducting wastewater surveillance to mitigate the impact of a second wave of cases. For example, in Altamonte Springs, Florida, elevated viral concentrations in wastewater represented a leading indicator of emerging cases. This was communicated to local healthcare providers, hospitals and assisted living facilities to give them advance warning and a chance to implement personal protective equipment protocols and strengthen testing capabilities [42].

At the building level and in congregate living spaces, there are multiple reports of wastewater surveillance identifying asymptomatic or pre-symptomatic individuals prior to clinical testing, allowing for swift isolation and preventing further spread of outbreaks [43]. After registering a high count of nursing home fatalities at the beginning of the pandemic, France deployed widespread wastewater testing in nursing homes during the summer of 2020 to prevent clusters of cases in this high risk population [44]. It is estimated that several outbreaks were prevented in senior care facilities due to the rapid implementation of mass polymerase chain reaction (PCR) testing of residents and staff, with subsequent isolation of infected individuals [45].

### Monitoring disease spread and effectiveness of public health interventions

Beyond serving as an early warning system, wastewater surveillance can also be applied to monitor disease spread and evaluate the effectiveness of public health interventions. In the early stages of the COVID-19 pandemic, estimating the true scope of disease penetration using clinical data remained a challenge for large segments of the population as asymptomatic infected individuals continued to drive transmissions and clinical testing remained limited. Comparing wastewater analysis with reported cases helped reveal the extent to which diagnostic testing was underestimating the true incidence of disease, signalling the need to continue investing in public health infrastructure while scaling up testing [46, 47].

Another use of wastewater-based epidemiology is to provide an independent estimate of community-level disease activity and trends, offering public health officials an additional view of infection in a population such as indicating whether COVID-19 activity is on the rise or diminishing [48]. For example, a collaborative effort between the Utah Department of Environmental Quality, the Utah Department of Health, and academic laboratories allowed for wastewater surveillance across the state over several months [49]. In July 2020, reported COVID-19 cases were declining. However, at the same time, the number of people undergoing individual diagnostic testing had also decreased, raising the possibility that the reported declining case rates were artifacts of clinical testing efforts. In parallel, wastewater surveillance data indicated a decline in viral concentrations in sewage, confirming that the decreasing trend in COVID-19 disease activity observed in clinical testing data was accurate [49].

Wastewater data can thus be used alongside complementary data sources, such as clinical data, to inform various public health decisions and can help gauge the effectiveness of policy interventions. For example, a decrease in SARS-CoV-2 levels in response to stay at home orders may prompt governments to begin a structured and phased reopening plan. During a reopening, trends in SARS-CoV-2 levels in wastewater may help gauge the speed of reopening or the need to scale back. In February 2021 in New Zealand, case data was coupled with wastewater data to decide whether to extend a lockdown after three positive cases were detected. Negative results from wastewater PCR testing at the municipal level allowed government officials to rule out community spread of infections and defined it rather as a contained and traceable cluster of individuals [50].

Lastly, in the current phase of the pandemic in the United States, with a partially vaccinated population and COVID-19 testing demand projected to decline, monitoring SARS-CoV-2 viral concentrations in wastewater can lend credence to case numbers. Case counts might be low due to high vaccination rates, high natural immunity, or insufficient testing; wastewater surveillance data can offer additional clues to distinguish between the underlying causes. This information can then be used to deploy spatially targeted vaccination programs to suppress transmission and achieve local containment of COVID-19 disease [51].

### WBE as a complement to genomic epidemiology

Wastewater can serve as a useful complement to existing genomic epidemiology efforts. In the ongoing COVID-19 pandemic, multiple variants of concern (e.g., alpha, delta, lambda, mu) have emerged since December 2020 [52, 53]. These variants were identified through sequencing of the genome in clinical specimens, and were quickly followed by the development of multiple assays to detect and monitor variants in wastewater [54]. Individual patient clinical sequencing remains the standard method to identify novel mutations and lineages associated with phenotypic differences (e.g. increased mortality, increased transmission), but this approach is difficult to scale across large populations and implement as a routine real-time monitoring tool. Wastewater samples include a mixed set of circulating variants that can complement clinical genomic surveillance. Novel mutations and lineages can be identified via clinical sequencing and rapidly deployed through WBE by the development of targeted assays. In this paradigm, novel variants identified in the clinical setting can quickly become monitored across entire populations. However, the presence of multiple viral lineages in wastewater, combined with the lower viral concentrations of wastewater samples relative to clinical samples, presents methodological challenges that require further research and development [55].

## Implementation considerations: Wastewater testing at different scales

**Fig 1.**
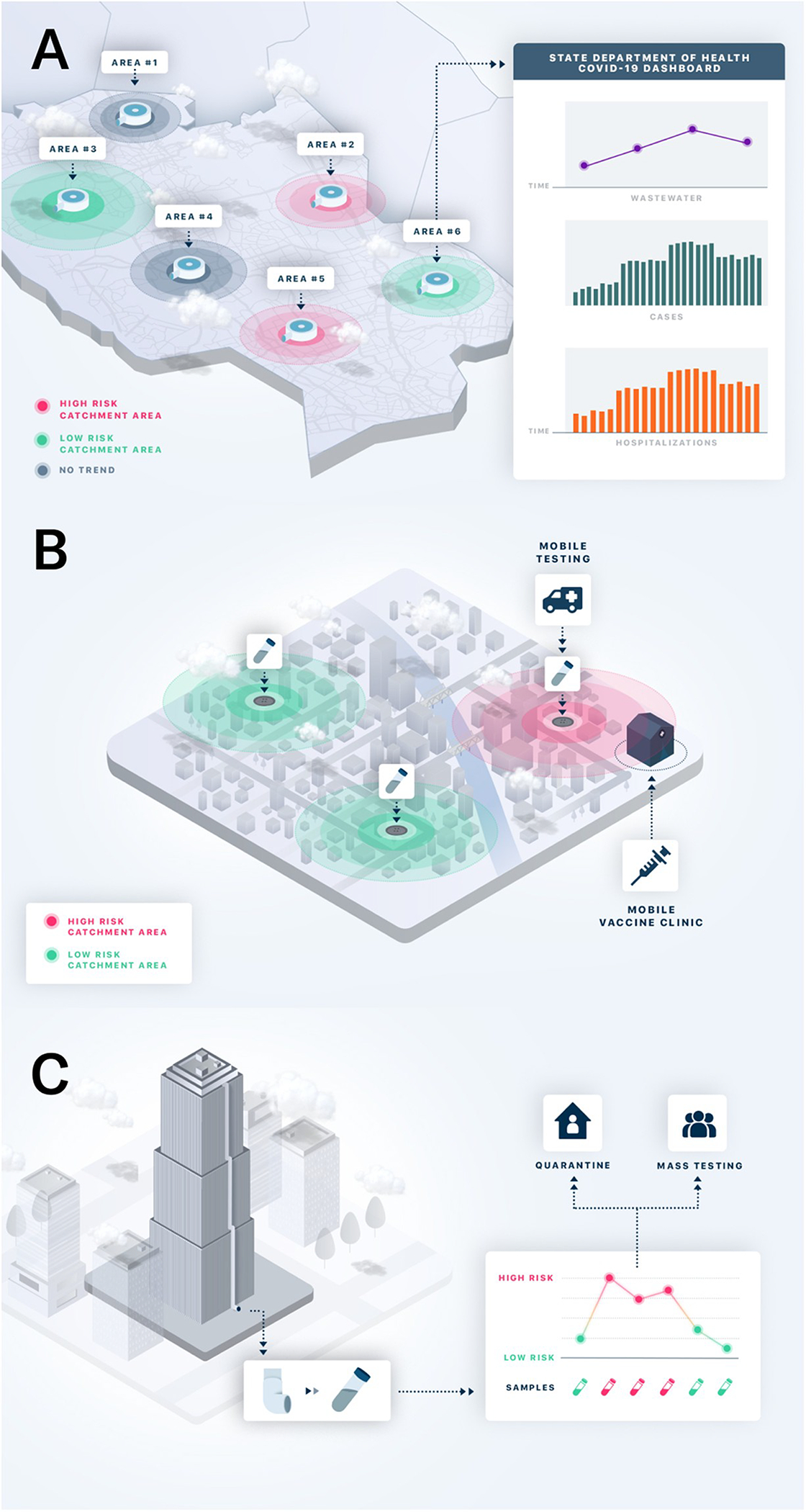
Implementation of WBE at three geographic scales: (A) wastewater treatment plant level, (B) neighborhood level, and (C) building level. With permission from Biobot Analytics. https://doi.org/10.1371/journal.pgph.0000061.g001

While most WBE efforts take place downstream at the wastewater treatment plant, leveraging the collection source from individual buildings (upstream) to neighborhood manhole access sites can lend different geographic precision to wastewater-based epidemiology (Fig 1). While all wastewater sampling scales yield viral concentrations, the degree of resolution of these sampling scales have different logistical and data considerations that may inform public health decisions.

**Fig 2.**
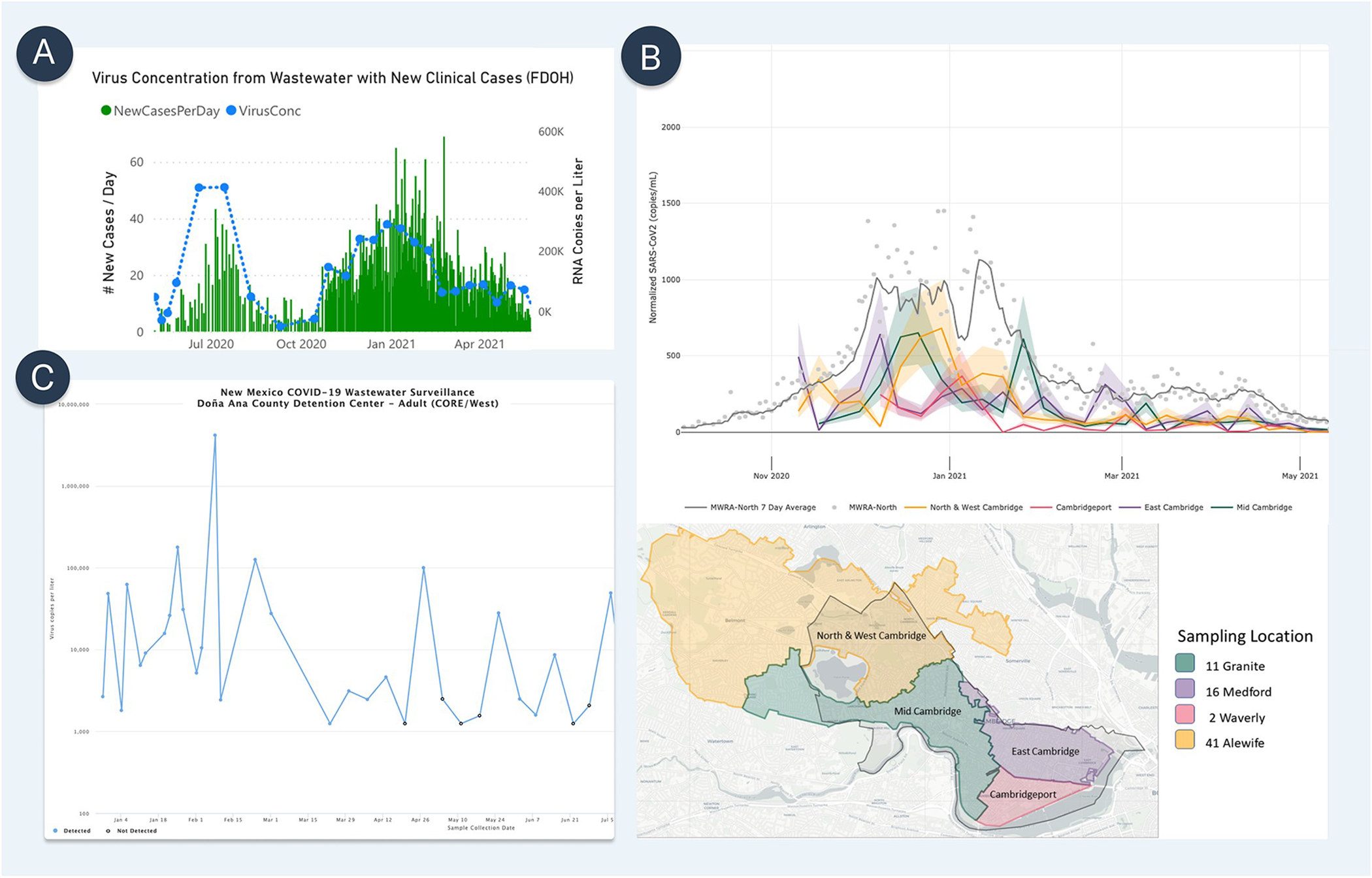
Examples of wastewater surveillance data visualization. (A) Wastewater treatment plant level: Wastewater Surveillance dashboard by the Loxahatchee River District, FL. Wastewater viral concentrations (blue dots) are compared against clinical case data (green bar). Screenshot taken by the authors on June 12, 2021, from https://loxahatcheeriver.org/wastewater-surveillance/. (B) Neighborhood level: Weekly Municipal Wastewater Sampling Data by Cambridge COVID-19 Data Center, Cambridge, MA. Samples are collected from four manholes within the City of Cambridge, which together cover the whole city. Wastewater viral concentrations at the four upstream catchments (orange, red, purple. green) are compared against each other and against a downstream catchment (gray) that covers Greater Boston. Screenshot taken by the authors on May 5, 2021, from https://cityofcambridge.shinyapps.io/COVID19/?tab=wastewater. (C) Building level: New Mexico Wastewater Surveillance System Data Dashboard, New Mexico Environment Department, NM. The line graphs represent the amount of the virus measured in wastewater samples collected at the correctional facility through time (blue). The current level of concern (bottom left) is based on the most recent sample while the current trend (bottom right) is based on the three most recently collected samples at each facility. Increasing and decreasing trends are flagged when there is an order of magnitude (or 10-fold) change in measurements. Screenshot taken by the authors on July 27, 2021 https://www.env.nm.gov/wastewater-surveillance-system-data-dashboard/. https://doi.org/10.1371/journal.pgph.0000061.g002

In the following sections, we critically review key considerations surrounding the decision to implement wastewater surveillance and operational factors in conducting WBE at three geographic sampling scales: the wastewater treatment plant level, neighborhood level, and building level (Fig 2, Table 1).

### Wastewater treatment plant level

Most wastewater treatment plants in the United States regularly collect and analyze wastewater samples to monitor water quality, making access to sewage at this level cost efficient and practical. For COVID-19 analysis, both untreated wastewater and primary sludge samples from wastewater treatment plants can be analyzed to measure viral concentrations [56, 57]. Longitudinally, viral concentrations can be mapped to represent trends of infections estimating and projecting new clinical COVID-19 cases or resurgence of disease in a large catchment area (Fig 2A) [29, 46, 58]. From a policy perspective, teams leading public health emergency preparedness and response activities can utilize wastewater surveillance data to anticipate potential surges of clinical cases and allocate resources strategically around a city, especially in areas where the need for medical care could outweigh the healthcare system capacity.

As an example, the Cambridge Public Schools in the City of Cambridge, Massachusetts, used wastewater surveillance data to design its back-to-school reopening strategy from September to December 2020 and included wastewater viral concentrations as one of the three metrics to track to safely operate and open their public schools [59, 60]. The Cambridge Public Schools system used data from samples taken at the Deer Island wastewater treatment plant, which services the entire greater Boston metropolitan area including the City of Cambridge. Rising levels of COVID-19 in wastewater at Deer Island would trigger the public school system to shift to remote learning district-wide. Cambridge Public Schools employs teachers and support staff from all over the greater Boston region. Understanding metropolitan-level COVID-19 infection therefore may suggest risk of infection among key teachers and staff within Cambridge Public Schools.

One limitation associated with city-level wastewater downstream sampling is the lack of granularity of results. Due to the aggregate nature of sewage at the wastewater treatment plant level, data readouts do not offer insight into intra-city variability, specific high-risk communities or sociodemographic factors that may drive infection rates.

### Neighborhood level

The ability to access and measure SARS-CoV-2 titers upstream from major wastewater treatment plants allows for increased spatial granularity around the scale of infection penetration in specific neighborhoods (Figs 1B and 2B).

At this level of sampling resolution, wastewater-based epidemiology can provide estimates of neighborhood burdens of infection. While neighborhood level wastewater-based surveillance is not a replacement for in-person testing, comparing wastewater results with clinical testing data on coverage and positivity rate can help officials see where gaps may exist with in-person testing, and may prompt addition of COVID-19 prevention and treatment resources. For example, in New Castle County, Delaware, in the Summer of 2020, the County Executive deployed wastewater testing in 12 different locations throughout the county as part of their COVID-19 response strategy [61]. By gathering this neighborhood-level data in a county of over 550,000 people, New Castle County public health experts were able to effectively position mobile testing units in the areas of significant need. When a spike in viral load was detected in a neighborhood’s wastewater, a mobile testing unit was quickly re-positioned in that area.

Similarly, in Guadalupe, Arizona, where COVID-19 testing capacities were limited, the mayor had challenges in assessing the spread of the virus through her community and struggled to access relevant COVID-19 data from regional authorities [62]. The nearby City of Tempe had partnered with a local university to test wastewater for COVID-19, and the program included sampling at points which isolated contributions of different neighborhoods to the city’s overall sewage [62]. Through this arrangement, they were able to detect high levels of SARS-CoV-2 in the sewage from the small community of Guadalupe, signalling that there was ongoing community spread of the virus. Leveraging this data set at this level of granularity, the mayor was able to advocate for her town and get the financial resources necessary to try and contain the spread of the virus in her community [62].

Neighborhood level sampling is also important for monitoring the reintroduction or resurgence of an infectious disease. Current wastewater analysis methods allow for the detection of one infected person in a population of thousands.^14, 28, 29^ In later stages of the ongoing COVID-19 pandemic, neighborhood level sampling, or sampling from wastewater treatment plants that serve small communities, will be key to ensure that any resurgence of disease is localized and quickly contained. Sampling at the neighborhood level is particularly useful at the beginning of the pandemic and in the recovery phases of the pandemic, when vigilant, cost effective and sustainable monitoring is required to monitor for resurgence of cases.

However, neighborhood level sampling has its own set of challenges. Knowledge of wastewater networks and geographic information system (GIS) analysis is needed to properly select a targeted sampling location. A sewage collection area is defined by the sewer network and may not exclusively cover the area of interest. Sampling from municipal manholes, often located on city streets, requires coordination and time from the Department of Public Works. While more consistent than building-level surveillance, the ebbs and flows of individuals through a community may also affect results at this level. Modeling of population influx and correction in sampling frequency and duration can mitigate these confounders to the data.

### Building level

At its highest geographic resolution, wastewater analysis can be applied to an individual building or business complex (Figs 1c and 2c). By collecting samples directly from a sewer pipe in a building or from a sewer access site outside a building, upstream sampling can include all residents of a single business or apartment complex. Applied to commercial office space, schools, or congregate living settings (e.g., nursing homes, college dormitories, correctional facilities) building-level wastewater analysis can detect the presence or absence of SARS-CoV-2 within the micro-community of occupants of a given building [63, 64]. Since wastewater at the building-level is an aggregate of all the human waste of the occupants of the building, it represents a cost-effective alternative to testing every single person. These insights can be applied to lend greater precision around the decision to test or quarantine the entire group of individuals living or working in a building. Frequent, even daily, sampling is likely particularly relevant in transient settings (such as corporate buildings, universities, and schools) where the probability of an infected building inhabitant depositing fecal matter in the toilet system of a given building is unpredictable. Increasing the sampling frequency therefore increases the chance of catching fecal matter from infected individuals when they use the restroom. A positive result for SARS-CoV-2 from building wastewater testing can prompt additional actions with consequences on the need to individually test occupants of the building, syndromic surveillance, or changes to occupant/visitor policies.

Implementation of wastewater surveillance at the building level started in the early days of the pandemic and in the US, the first notable successful application was from the University of Arizona. As students returned to campus for the Fall semester in 2020, the University of Arizona prevented an outbreak on campus when regular testing of sewage samples from 20 campus buildings revealed traces of the virus in a dormitory. This was followed by PCR testing of all students the next day which identified two positive cases with asymptomatic SARS-CoV-2 infections. These individuals were subsequently isolated, contract tracing was undertaken, and no further infections were associated with those cases first signalled through wastewater surveillance [43]. In correctional facilities, the presence of vulnerable populations in physically constrained environments has also prompted the use of wastewater analysis as an early warning tool to prevent outbreaks [33]. Because the wastewater sample is an aggregated readout of the infection status of all individuals who use a building’s restroom facilities, wastewater testing results may miss the presence of infected individuals if they did not have a bowel movement on-site on the sampling day. Therefore, building-level sampling runs the risk of creating a false sense of security if negative results are interpreted as the definitive absence of infected individuals on site.

## Limitations

Unlike other public health data sets, such as clinical data, wastewater surveillance data does not rely on counting the number of individuals with positive test results or specific symptoms. Instead, it is composed of data with a higher degree of variability. Therefore, it may not be as precise by nature and should not be relied upon solely for making public health decisions and policy changes. It remains nonetheless a powerful adjunctive tool to contextualize other key sources of public health data, like our current COVID-19 pandemic has demonstrated.

For wastewater-based epidemiology to live up to its full potential of addressing health equity concerns by representing all members of a society in its data, sewer networks would need to cover all geographic areas, including remote locations, to reach all individuals. However due to infrastructure deficiencies, many people live in communities not connected to a sewer network. In the US, 20% of households are not served by municipal sewage collection systems and are therefore not accounted for in WBE data sets [32]. This proportion is even higher in low resource settings globally. This physical infrastructure limitation introduces a selection bias regarding which populations are represented under a WBE approach and should be taken into account.

In addition, sampling at the wastewater treatment plant level WBE has been the most common wastewater sampling scale since the start of the pandemic, mainly due to its practicality and cost-efficiency. However, the lack of granularity of data associated with city-wide sampling prevents the understanding of heterogeneous risks within the catchment areas which may provide clues to drivers of infections. This in turn, also limits the efficacy of using trends data from city-level sampling for targeted interventions.

While neighborhood-level sampling has the overall advantage of providing more granularity with results, the occasional misalignment observed between wastewater catchment areas and public health reporting jurisdictions may complicate the comparison of wastewater data and reported cases to deploy resources to cover a specific population. Lastly, at the building level, in comparison to larger catchment areas, the transit of individuals in and out of buildings creates unpredictability of whether occupants will contribute human waste to the building-level wastewater network, which may limit data interpretation and actionability.

## Future frontiers

Multiple areas of scientific research will further advance the utility of wastewater-based epidemiology. First, patterns of fecal shedding of SARS-CoV-2, such as the percentage of infected individuals that shed the virus in feces and the dynamics of fecal shedding throughout the stages of infection, need to be better characterized [40, 65–67]. This research will improve estimates of the burden of COVID-19 disease in a community, and the likelihood of detecting infected individuals [68, 69].

Second, wastewater data originates from a complex heterogeneous matrix, which can complicate data interpretation. For example, there can be varying flow rates of sewage in cases of rain which lead to dilution of wastewater samples, or different sampling schedule and duration. To address the dilution challenge, normalization of the wastewater viral concentration with the human fecal indicator Pepper mild mottle virus (PMMoV) has helped reduce noise in wastewater data sets [46]. Normalization methods are still evolving to facilitate wastewater data interpretation across time and space [70, 71].

Third, wastewater sampling matrix type (e.g., raw wastewater, sludge), methods (automated composite samplers versus grab sampling), and collection duration (e.g., 24-hour composite sample) may also vary depending on the application of WBE as well as the setting, and the resources available. Further investigation is needed to define how these different parameters of wastewater sampling impact data interpretation. Lastly, as the field further progresses, more research is needed to refine our understanding of wastewater data interpretation and the ability to discern which wastewater signals call for which types of public health action.

There are also important areas for future policy work. First, wide scale adoption of wastewater surveillance amongst public health officials is still not established due in part to limited understanding of the characteristics of this new data set. Public health guidelines on the interpretation of wastewater data for public health action need to be developed and training programs implemented in departments of public health across levels of government. This will help build familiarity and support for WBE among public health cadres to establish wastewater surveillance as a permanent pillar of emerging epidemic and pandemic preparedness and response [72]. A few barriers will need to be addressed before there is wide-scale adoption of wastewater surveillance and response infrastructure. Secondly, while the physical aggregation of human waste in sewer networks provides natural de-identification and protection of privacy, concerns over stigmatization of communities exist and the ethical and privacy limitations of wastewater surveillance have emerged as an area of focus. These concerns should be addressed when developing ethical standards regarding wastewater data collection, reporting, interpretation and use to support the wide scale adoption of wastewater monitoring across geographic scales^24^. Finally, irrespective of the level of sampling, wastewater sampling requires a commitment of resources by public and private actors to cover the material costs associated with laboratory equipment and analysis as well as human resource time necessary for the implementation and monitoring of WBE.

## Conclusion

Wastewater and sewer infrastructure are present throughout the United States. Leveraging these systems at various geographic scales for epidemiological surveillance can provide important insights into the spread of infectious disease and biological pathogens within a population. Applied to the COVID-19 pandemic, mapping the distribution of SARS-CoV-2 infection throughout a community has provided evidence to justify deployment of key public health measures. While unlikely to inform all aspects of pandemic preparedness and response on its own, wastewater-based epidemiology is a useful tool that when used in combination with clinical testing and case reporting, can be used to provide an early warning for new biological threats. WBE can also monitor the evolution of a pathogen amongst communities, identify hotspots of infection, and evaluate the effectiveness of public health interventions.

Whether monitoring is deployed at a building, neighborhood or municipal level, wastewater can provide valuable information and support confirmation of trends observed through clinical data. Future implications go beyond the current COVID-19 pandemic; WBE has emerged as a useful tool to control infectious disease outbreaks and monitor population-level biomarkers of human health. As it becomes an important pillar of pandemic and epidemic preparedness and response systems, wastewater-based epidemiology can support public health policy as an established surveillance tool for emerging infectious diseases and biological threats.

## Figures and Tables

**Table 1.** Overview of wastewater geographic scales of sampling, utility of results, public health implications and limitations.

Sampling level	Utility of results	Public Health decisions	Limitations
Wastewater treatment plant (Downstream)	Trend analysis	Implementation of population level interventions (e.g., stay at home orders) and impact evaluation	Sampling at large scale does not provide insight into intra-city variability; does not offer insight into sociodemographic factors that may drive heterogeneous infection rates.
	Independent estimate of population-level disease activity		
	Indicator of disease re-emergence		Downstream dilution effects
Neighborhood	Trend analysis	Strategic deployment of mobile testing units, targeted social distancing messaging, vaccination sites	Sewage collection area is defined by sewer network and may not exclusively cover the area of interest.
(Upstream)	Relative burden of disease activity of a neighborhood within a larger community		
	Indicator of disease re-emergence	Community-level COVID-19 restrictions	Labour costs, logistics associated with collecting samples from city manholes.
			Data needs to be interpreted considering influx to, and out of, the catchment area
Building (Upstream)	Qualitative presence or absence of disease on site Outbreak indicator	Quarantine of building occupants Mass testing of building occupants	Result only provides a readout of the infection status of individuals who have used the building restroom facilities

https://doi.org/10.1371/journal.pgph.0000061.t001
